# Preparation and immunological effects of a bivalent subunit candidate vaccine based on the PCV-Cap-2-3 gene engineered from baculovirus

**DOI:** 10.3389/fvets.2026.1849112

**Published:** 2026-07-15

**Authors:** Yi Liu, Tong Hao, Chengyu Wang, Xiaoxin Chen, Anqi Ju, Jianqiang Liu, Hongtao Wang, Lichun Zhang, Rui Du

**Affiliations:** 1 Jilin Academy of Agricultural Sciences, Changchun, China; 2 Ginseng and Antler Products Testing Center of the Ministry of Agricultural PRC, Jilin Agricultural University, Changchun, China; 3 College of Animal Science and Technology, College of Veterinary Medicine, Jilin Agricultural University, Changchun, China; 4 Department of Veterinary Medicine, College of Agriculture, Yanbian University, Yanji, China

**Keywords:** PCV-Cap-2-3 gene, porcine circovirus type 2, porcine circovirus type 3, recombinant baculovirus, subunit vaccine

## Abstract

**Introduction:**

Porcine circoviruses (PCVs), belonging to the genus *Circovirus* in the family Circoviridae, are prevalent worldwide and pose a serious threat to the pig industry. Although vaccines against PCV2 are available, PCV3 vaccine development, prevention strategies, and surveillance for co-infections remain limited, leading to delayed outbreak responses. Therefore, the aim of this study was to develop a bivalent subunit vaccine targeting the prevalent strains of PCV2 and PCV3.

**Methods:**

Recombinant baculoviruses, Baculovirus-PCV2-Cap and Baculovirus-PCV3-Cap, were constructed to express the PCV2-Cap and PCV3-Cap proteins, respectively. Additionally, a recombinant baculovirus, Baculovirus-PCV-Cap-2-3, was engineered to co-express both proteins. Using GEL02 as the subunit vaccine adjuvant, purified proteins were formulated at an antigen concentration of 30 μg/mL. PCV2-Cap, PCV3-Cap, and PCV-Cap-2-3 subunit vaccines were prepared and administered to 28-day-old piglets.

**Results:**

Serological analysis showed that all vaccines induced the production of PCV-specific and neutralizing antibodies in pigs. Lymphocyte proliferation assays demonstrated that the PCV-Cap-2-3 subunit vaccine successfully induced cellular immune responses. Post-vaccination challenge protection tests showed that, following PCV2 and PCV3 challenge, the PCV-Cap-2-3 subunit vaccine group exhibited reduced pathological damage in organs compared with the PBS control group.

**Discussion:**

Overall, these results indicate that the developed vaccine effectively protected against both PCV2 and PCV3. The protective performance of this vaccine was comparable to that of commercial PCV2 vaccines, inducing robust immune responses in vaccinated pigs and showing potential for further development.

## 1 Introduction

Porcine circoviruses (PCVs) are very small, non-enveloped animal viruses possessing a circular single-stranded DNA genome of approximately 2 kb. They belong to the genus *Circovirus* within the family Circoviridae, which are prevalent worldwide and pose a serious threat to the pig industry (1). Four PCV types, namely porcine circovirus types 1–4 (PCV1–4), have been reported. PCV1 was initially identified as a contaminant in serially passaged porcine renal cells, and antibodies against it are highly prevalent in pig herds worldwide. Unlike PCV1, PCV2 is pathogenic to pigs and can lead to post-weaning multisystemic wasting syndrome (2–4). Additionally, PCV2 is the causative agent of PCV-associated diseases (PCVADs) (5). PCV2 can be categorized into eight subtypes (PCV2a–PCV2h), among which PCV2d is currently predominant in China (6, 7).

PCV3 was first identified in pigs with porcine dermatitis and nephrotic syndrome (PDNS) in the United States in 2016 (8). However, its pathogenic role and whether disease-associated injury occurs are still under investigation. Several PCV3 genotyping methods are available, enabling classification of PCV3 into PCV3a, PCV3b, and PCV3c genotypes based on conserved differences in amino acids at positions 24 and 27 of the coat protein (9). PCV4 was first detected in Hunan Province, China, in 2019 and has since been reported in several regions of the country in recent years (10). Its potential association with severe clinical diseases such as PDNS, respiratory symptoms, and intestinal manifestations has been suggested (11). With increasing research into PCV2 and PCV3, co-infections have been identified at rates of 3.4–11.0% (3). Although a vaccine for PCV2 has already been marketed, vaccine development for PCV3 remains in its early stages, and prevention and control strategies are relatively limited. Furthermore, current surveillance and diagnostic methods remain inadequate for co-infections, resulting in delayed outbreak responses. Therefore, combined prevention and control strategies against PCV2 and PCV3 are urgently needed.

The PCV genome contains two major open reading frames (ORFs), ORF1 and ORF2. The PCV2 capsid protein (Cap), encoded by ORF2, is the main structural protein of the virus. It contains antigenic epitopes capable of inducing specific neutralizing antibodies in the host and therefore represents a promising target for recombinant vaccine development (12). To address the dual challenges posed by the widespread prevalence of the PCV2d genotype and the current lack of commercially available vaccines against PCV3, this study aimed to use a baculovirus vector to express PCV2 Cap and PCV3 Cap proteins and to develop a bivalent subunit vaccine for the simultaneous prevention of PCV2 and PCV3 infections. The development of the PCV2-PCV3 bivalent subunit vaccine may provide an effective strategy for controlling these infections in commercial pig herds.

## 2 Materials and methods

### 2.1 Construction of recombinant baculovirus

**Figure 1:**
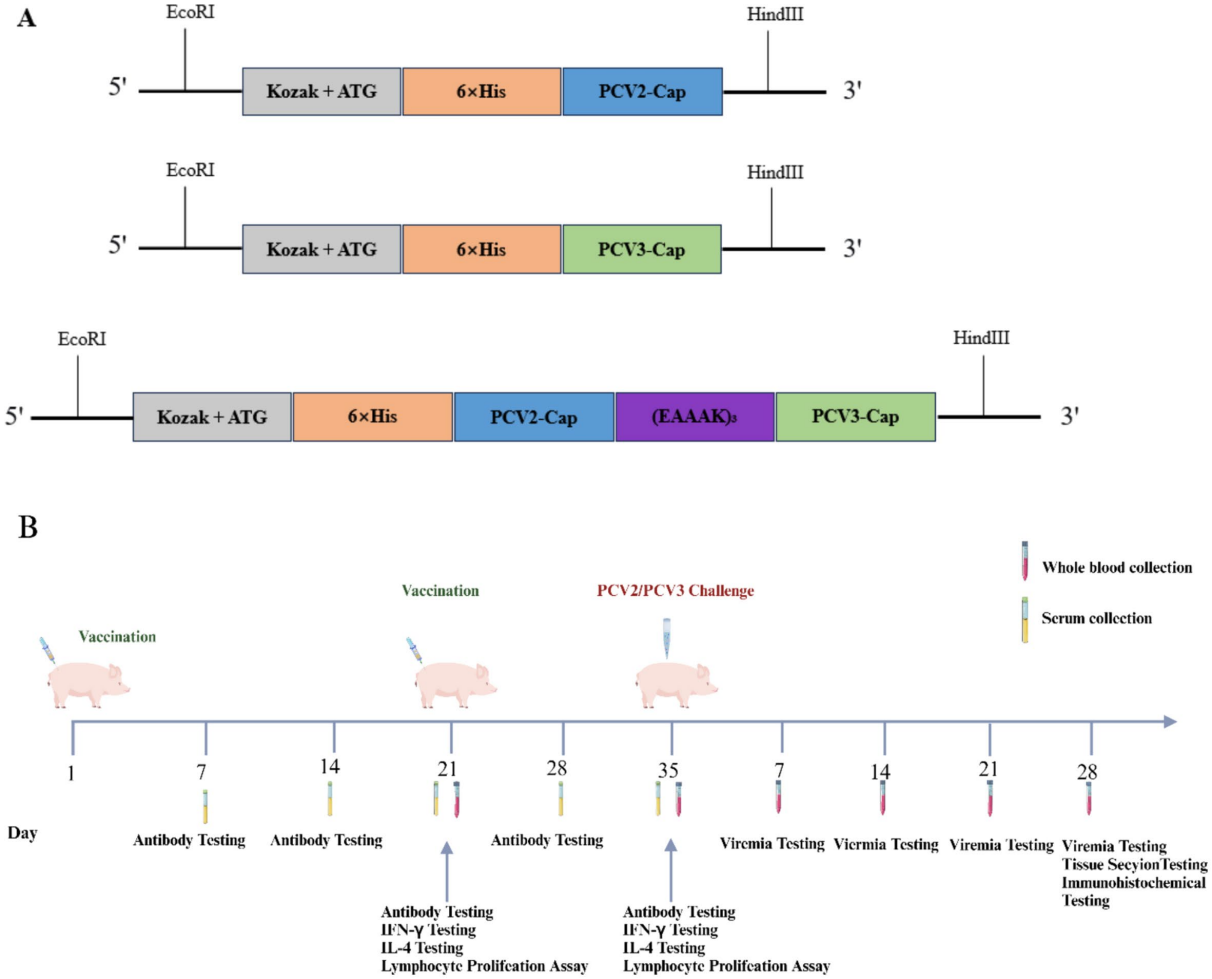
Flowchart of recombinant expression vector construction and animal experimental design with **(A)** schematic construction of the recombinant expression vectors, and **(B)** experimental design for pig immunization and viral challenge.

The PCV2-Cap and PCV3-Cap genes isolated in our laboratory were codon-optimized according to the codon usage preference of *Spodoptera frugiperda* (Sf-9) cells. Subsequently, a 6 × His tag and a Kozak sequence (GCCACC) were introduced at the 5′ ends of the optimized PCV2-Cap and PCV3-Cap sequences. In the fusion construct, the PCV2-Cap and PCV3-Cap sequences were linked using a rigid linker peptide, (EAAAK)₃. The nucleotide sequence encoding (EAAAK)₃ was GAG GCT GCA GCT AAA GAG GCT GCA GCT AAA GAG GCT GCA GCT AAA. The respective sequences for each group were inserted into the multiple cloning site of the pFastBacI vector between the EcoRI and HindIII restriction sites (Thermo Scientific, Shanghai, China). Synthesis of these optimized constructs and their cloning into the pFastBacI vector were performed by Comate Bioscience Co., Ltd. The resulting plasmids were designated as Baculovirus-PCV2-Cap, Baculovirus-PCV3-Cap, and Baculovirus-PCV-Cap-2-3, respectively (Figure 1A).

### 2.2 Construction of recombinant bacmid transposons

Plasmids were validated via restriction enzyme digestion with EcoRI and HindIII and DNA sequencing. Recombinant and empty vector plasmids were transformed into DH10Bac competent cells, with the empty vector plasmid pFastBacI used as a negative control. Sf9 cells were cultured at 27 °C in Sf-900 II SFM (Gibco, Grand Island, NY, USA). When the Sf9 cells reached optimal growth, viability was assessed using trypan blue staining. Cells with viability ≥95% were seeded into six-well plates at 1 × 10^6^ cells/mL. Transfection was performed when the cells reached 80–90% confluency. On days 3–5, after transfection of recombinant plasmids in each group, observe cellular changes. When the cells become enlarged and rounded, and intracellular granules are clearly visible, collect the viral supernatant and designate them as Baculovirus-PCV2-Cap, Baculovirus-PCV3-Cap, Baculovirus-PCV-Cap-2-3, and Baculovirus-NC (with the empty vector plasmid pFastBacI).

### 2.3 Amplification of recombinant baculovirus

Cells were seeded into 25 T flasks at 2 × 10^6^ cells/mL. After the cells had settled, 10 μL of P0 Baculovirus-PCV2-Cap, P0 Baculovirus-PCV3-Cap, or P0 Baculovirus-PCV-Cap-2-3 viral solution was inoculated into each flask. Sf9 cells showed cytopathic effects approximately 3 days post-infection. On days 4–5, the cells exhibited enlargement, rounding, and increased granularity. The harvested viral solutions were designated as P1 Baculovirus-PCV2-Cap, P1 Baculovirus-PCV3-Cap, and P1 Baculovirus-PCV-Cap-2-3, respectively, and stored at 4 °C in the dark for short-term use. Viral titers were determined for each sample.

### 2.4 Recombinant protein expression and purification

Sf9 cells were mass cultured, and when the cell density reached approximately 1–2 × 10^6^ cells/mL, the cells were infected with recombinant baculovirus at a multiplicity of infection of 1. At 72–96 h post-infection, when approximately 80% of the cells exhibited distinct cytopathic effects, the cells were harvested, washed with cold PBS, lysed, and centrifuged to obtain the supernatant. Recombinant protein expression was confirmed by polyacrylamide gel electrophoresis (PAGE). The recombinant proteins were purified from the Sf9 cell supernatant using a HisTrap HP 5 mL column (Cytiva, MA, USA) based on His-tag Ni-NTA affinity chromatography. The target proteins were eluted using a buffer containing 20 mM Tris–HCl (pH 8.0), 150 mM NaCl, and 300 mM imidazole. The concentration of the purified protein was determined using the BCA Protein Assay Kit (Takara, Beijing, China).

### 2.5 Indirect immunofluorescence

A coverslip was placed in a 24-well plate under aseptic conditions, and Sf9 cells were seeded onto the coverslip at a density of 1 × 10^6^ cells/mL. After the cells were well-attached and reached 70–80% confluency, the cell culture medium was removed. The cells were infected with wild-type baculovirus (Baculovirus-NC) and the recombinant baculoviruses Baculovirus-PCV2-Cap, Baculovirus-PCV3-Cap, and Baculovirus-PCV-Cap-2-3. At 72–96 h post-infection, when approximately 80% of the cells exhibited cytopathic effects, the cell culture medium was discarded. The cells were then fixed with 4% paraformaldehyde at 18–24 °C for 15 min and permeabilized with 0.1% Triton X-100 in PBS for 10 min at ca. 25 °C. The slides were blocked with 5% BSA at 37 °C for 2 h, after which the blocking solution was discarded. The cells were incubated overnight at 4 °C with an mouse anti-His tag primary antibody (1:1,000, Bioss, Beijing, China). After washing three times with PBS, the cells were incubated with Goat Anti-Mouse IgG Cy5 (1:1,000, Bioss) in the dark for 1 h. The fluorescent secondary antibody solution was discarded, an appropriate amount of antifluorescent quencher was added, and the slide was examined and photographed using a laser confocal microscope.

### 2.6 Western blot analysis

PCV2-Cap and PCV3-Cap proteins (10 μL) were analyzed by western blotting. The primary antibody used for detection was a PCV2-Cap/PCV3-Cap rabbit polyclonal antibody (1:2,000 dilution, Bioss), followed by a horseradish peroxidase-labeled goat anti-rabbit secondary antibody (1:5,000 dilution, Bioss). For the detection of PCV-Cap-2-3, the primary antibody used was a mouse anti-His monoclonal antibody (1:2,000 dilution, Bioss) was used, and the secondary antibody was an HRP-labeled goat anti-mouse IgG (1:5,000 dilution, GeneTex, Irvine, CA, USA).

### 2.7 Vaccine preparation

To compare the immunogenicity of the bivalent PCV-Cap-2-3 fusion protein with those of the monovalent PCV2-Cap and PCV3-Cap proteins, all vaccine formulations contained the same protein concentration (30 μg/mL). The adjuvant GEL02 (SEPPIC, France) was used at a final concentration of 10% (v/v) by mixing the antigen with a 20% (v/v) GEL02 stock solution at a 1:1 (v/v) ratio. The adjuvant was sterilized by filtration through a 0.22 μm filter prior to mixing. The aqueous phase (antigen buffer) was adjusted to pH 6 ± 0.2. To prepare the emulsion, the antigen was slowly added to the adjuvant while stirring, then the mixture was passed through a syringe 10 times by rapidly pushing and pulling the plunger. The emulsified vaccine was transferred into a sterile tube and assessed for stability by standing at 4 °C for 24 h (the emulsion was considered stable if phase separation was less than 10% of the total volume). The final formulation was stored at 4 °C until use.

### 2.8 Animal experiments

The animal study was approved by the Institutional Animal Care and Use Committee of the Jilin Academy of Agricultural Sciences (approval number: JNK20180831-05). The study was conducted in accordance with the local legislation and institutional requirements.

**Figure 2:**
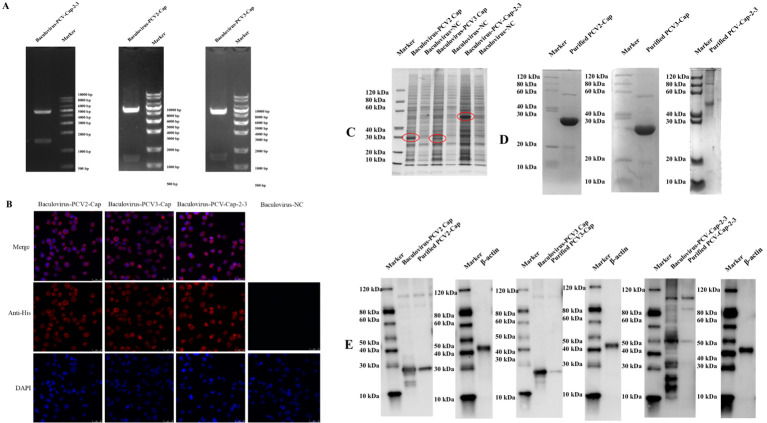
Recombinant baculovirus construction and protein expression. **(A)** Validation of recombinant plasmids Baculovirus-PCV2-Cap, Baculovirus-PCV3-Cap, and Baculovirus-PCV-Cap-2-3 via double-enzyme digestion analysis. **(B)** Indirect immunofluorescence assay confirmed the expression of Baculovirus-PCV2-Cap, Baculovirus-PCV3-Cap, and Baculovirus-PCV-Cap-2-3 in Sf9 cells. **(C)** SDS–PAGE analysis of recombinant proteins. **(D)** SDS–PAGE analysis of purified recombinant proteins. **(E)** Western blot analysis of recombinant proteins.

Healthy piglets were purchased from a large-scale farm in Qianguo County (Jilin Province, China). Before the experiment, all 28-day-old pigs tested negative for PCV2, PCV3, and PRRSV antigens and antibodies. Blood samples were collected from the anterior vena cava of the experimental pigs at 7, 14, 21, 28, and 35 days after the first immunization, and the serum was separated for humoral immune testing and cytokine detection. A secondary immunization was performed 21 days after the first immunization. On days 21 and 35 after the first immunization, peripheral blood lymphocytes were isolated for analysis of lymphocyte proliferation. Additionally, 35 days after the first immunization, pigs in each group were challenged with PCV2/PCV3. For the PCV2/PCV3 challenge, 2 mL of virus suspension was injected into the muscles at the back of the neck, and an additional 2 mL was administered intranasally. The PCV2 challenge dose was 10^6^ TCID_50_/mL. The PCV3 inoculum consisted of cell culture fluid obtained after five serial passages in primary porcine kidney cells in our laboratory, with a viral load of 4.0 × 10^7^ copies/mL. Blood samples were collected at 7, 14, 21, and 28 days post-challenge to detect viremia. At 28 days post-challenge, lung and inguinal lymph node samples were collected for histopathology and immunohistochemical analyses. The immunization doses and viral challenge groups are shown in Figure 2. The experimental groups included PCV2-Cap, PCV3-Cap, PCV-Cap-2-3 (bivalent subunit vaccine), PCV2-CV (Ingelvac CircoFLEX™, USA), and PBS (negative control). Each of the PCV2-CV, PCV2-Cap, and PCV3-Cap groups comprised five pigs, whereas each of the PBS and PCV-Cap-2-3 groups comprised 10 pigs.

### 2.9 Specific antibody assay

Enzyme-linked immunosorbent assay (ELISA) kits (SenBeiJia, Nanjing, China) for the detection of PCV2-Cap- and PCV3-Cap-specific antibodies were used to measure antibody titers at 7, 14, 21, 28, and 35 days post-immunization.

### 2.10 Neutralizing antibody detection

PK-15 cells were seeded into 96-well plates at a density of 1.5 × 10^5^ cells/mL. Serum samples were heat-inactivated (56 °C, 30 min) and serially diluted two-fold from 1:2 to 1:256 in MEM. Each dilution (100 μL) was mixed with an equal volume of virus solution containing 200 TCID₅₀/100 μL of PCV2 or PCV3 and incubated at 37 °C for 1 h. The following controls were included in each plate: (i) virus control: MEM instead of serum; (ii) cell control: MEM instead of both serum and virus; (iii) negative serum control: known PCV2/PCV3-negative pig serum; (iv) positive serum control: known PCV2/PCV3-positive pig serum. The mixture (100 μL) was then transferred to PK-15 cell monolayers in 96-well plates and incubated at 37 °C for 1 h to allow viral adsorption. After removal of the inoculum, cells were washed twice with PBS and cultured in maintenance medium for 48 h. The cells were then fixed with 4% paraformaldehyde for 10 min, permeabilized with 0.1% Triton X-100, and blocked with 5% BSA. Virus-infected cells were detected using mouse anti-PCV2-Cap or anti-PCV3-Cap antibodies (1:1,000; Bioss), followed by HRP-labeled goat anti-mouse IgG (1:5,000; Bioss). AEC (Solarbio, Beijing, China) substrate was added and incubated for 20 min. The positive wells were counted under a microscope (Leica, Wetzlar, Germany).

### 2.11 Lymphocyte proliferation assay

Peripheral blood lymphocytes were isolated from whole blood collected from immunized pigs using a porcine peripheral blood lymphocyte isolation kit (Solarbio, Beijing, China). Peripheral blood lymphocyte proliferation was assessed using an MTT assay kit (Beyotime, Shanghai, China). The absorbance was measured at 570 nm using a microplate reader (BioTek Instruments, USA), and the stimulation index (SI) was calculated.

### 2.12 Cytokine assay

Serum levels of IFN-γ and IL-4 were measured using ELISA kits (MeiMian, Jiangsu, China).

### 2.13 Statistical analysis

All results were analyzed using two-way ANOVA analysis of variance using GraphPad Prism 10.4.1. Tukey’s test was used to compare means when the treatment effect was significant (*p* < 0.05).

## 3 Results

### 3.1 Identification of recombinant transfer vectors

The three recombinant transfer vectors—Baculovirus-PCV2-Cap, Baculovirus-PCV3-Cap, and Baculovirus-PCV-Cap-2-3—were each verified by double digestion with EcoRI and HindIII. Each digest produced a vector backbone of approximately 4,700 bp and an insert of the expected size: 663 bp (PCV2-Cap), 723 bp (PCV3-Cap), or 1,532 bp (PCV-Cap-2-3), respectively (Figure 2A). After confirmation by restriction enzyme digestion and agarose gel electrophoresis, the three correctly constructed transfer vector plasmids were submitted to Comate Bioscience Co., Ltd. for sequencing. Sequencing analysis confirmed that the inserted fragments contained no unintended nucleotide mutations, and the deduced amino acid sequences matched the expected Cap protein sequences.

### 3.2 Verification of recombinant protein expression

Following fluorescent staining, Sf9 cells infected with the recombinant baculoviruses exhibited bright red fluorescence, whereas those infected with the wild-type Baculovirus-NC strain showed no fluorescent signal (Figure 1B), indicating successful expression of the recombinant proteins.

After ultrasonic disruption, the whole-cell lysates of Sf9 cells infected with recombinant baculovirus were analyzed by SDS–PAGE. The results showed multiple protein bands, as expected for crude lysates. However, in the Baculovirus-PCV2-Cap, Baculovirus-PCV3-Cap, and Baculovirus-PCV-Cap-2-3 groups, distinct bands of the expected molecular weights were observed (PCV2-Cap at 29 kDa, PCV3-Cap at 26 kDa, and PCV-Cap-2-3 at 55 kDa), whereas no such bands appeared in the Baculovirus-NC control group (Figure 2C, left). After affinity purification, SDS–PAGE analysis of the purified recombinant proteins revealed a single band of the expected size for each recombinant protein, with no significant background bands (Figure 2C, right).

Western blot analysis using β-actin as a positive control in whole-cell lysates detected target bands corresponding to the expected molecular weights of PCV2-Cap, PCV3-Cap, PCV-Cap-2-3, and β-actin (Figure 2E), confirming antigen-specific detection of the expressed proteins.

### 3.3 Antibody detection

**Figure 3:**
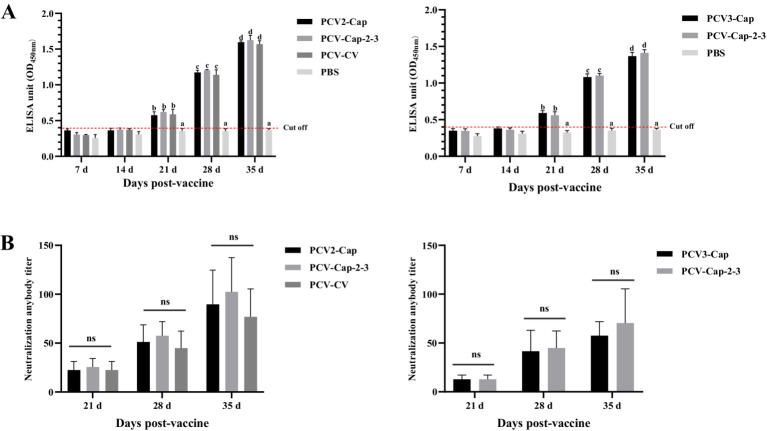
Evaluation of the immunogenicity of subunit vaccines. **(A)** Levels of PCV2-Cap/PCV3-Cap-specific antibodies elicited by subunit vaccine immunization. The assay detection limit was OD_450_ nm = 0.4 (dotted line). Values are presented as the mean ± standard error (SE) from five replicates. Values with different letters indicate significant differences (*p* < 0.05) among the groups; Values with the same letters indicate significant differences (*p* > 0.05) among the groups. **(B)** Neutralizing antibody titers against PCV2/PCV3 induced by subunit vaccine immunization. Values are presented as the mean ± standard error (SE) from five replicates, ns as not significant (*p* > 0.05) among the groups.

All experimental groups (PCV2-Cap, PCV3-Cap, and PCV-Cap-2-3) exhibited increased levels of PCV2-Cap- or PCV3-Cap-specific antibodies from 21 to 35 days post-immunization (*p* < 0.05), whereas those in the PBS group remained consistently negative. In PCV2-Cap antibody detection (Figure 3A), the antibody levels in the PCV2-Cap and PCV-Cap-2-3 groups were slightly higher than those in the PCV2-CV group (*p* > 0.05). In PCV3-Cap antibody detection (Figure 3A), no significant difference was observed between the PCV3-Cap and PCV-Cap-2-3 groups (*p* > 0.05).

These results indicate that the recombinant baculovirus-expressing PCV-Cap-2-3 subunit vaccine elicited PCV2-Cap antibody levels comparable to those elicited by the commercial vaccine, as well as inducing PCV3-Cap antibodies at levels similar to or slightly higher than those of the PCV3-Cap group (*p* > 0.05). Neutralizing antibody assays corroborated these findings. At 35 days post-immunization, PCV-Cap-2-3 induced the highest neutralizing antibody titers against PCV2 and PCV3, reaching titers of 1:102 and 1:70, respectively (Figure 3B).

### 3.4 Cellular immunity assays

#### 3.4.1 Lymphocyte proliferation assay

**Figure 4:**
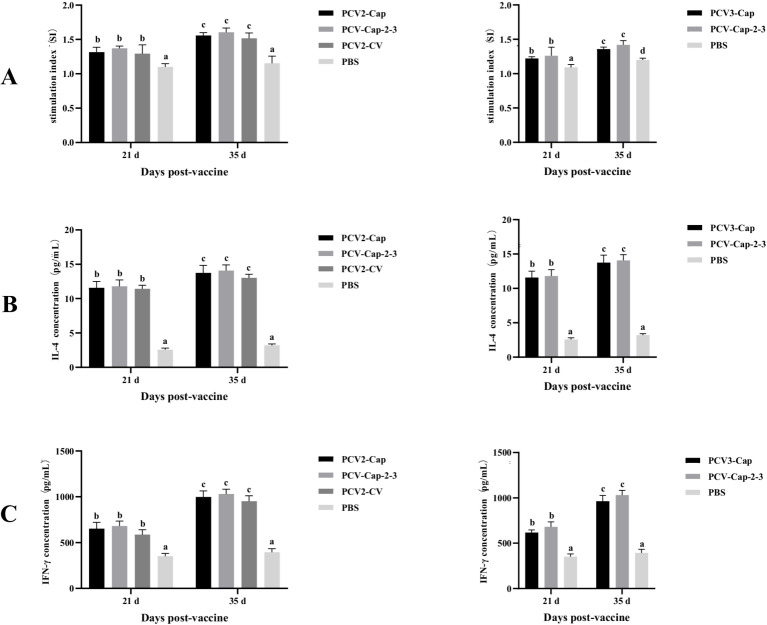
Detection of splenic lymphocyte proliferation and cytokine levels. **(A)** Splenic lymphocytes were isolated and stimulated with Con A, followed by PCV2 or PCV3 for 48 h, and proliferation was assessed using an MTT assay. **(B,C)** Levels of IL-4 and IFN-γ in lymphocyte supernatants were measured in the different groups after vaccination. Values are presented as the mean ± standard error (SE) from five replicates. Values with different letters indicate significant differences (*p* < 0.05) among the groups; Values with the same letters indicate significant differences (*p* > 0.05) among the groups.

At 21 and 35 days post-immunization, all experimental groups exhibited significantly higher PCV2/PCV3-specific lymphocyte proliferation responses than the PBS group (*p* < 0.05) (Figure 4A). The response levels observed at day 35 were higher than those at day 21 (*p* < 0.05). The PCV2-Cap and PCV-Cap-2-3 groups showed no significant difference from the PCV2-CV group in terms of PCV2-specific proliferation responses (*p* > 0.05). Similarly, the PCV3-Cap and PCV-Cap-2-3 groups exhibited no significant difference in terms of PCV3-specific proliferation responses (*p* > 0.05).

#### 3.4.2 Cytokine detection

All experimental groups exhibited significantly higher IFN-γ (Figure 4B) and IL-4 (Figure 4C) levels at 21 and 35 days post-immunization compared with the PBS group (*p* < 0.05). The levels at day 35 were higher than those at day 21 (*p* < 0.05). Cytokine expression in the PCV-Cap-2-3 group was marginally higher than that observed in the PCV2-CV group but not significantly so (*p* > 0.05) and was higher than that of both the PCV2-Cap and PCV3-Cap groups (*p* > 0.05). These results suggest that the PCV-Cap-2-3 subunit vaccine induces a cytokine response similar to or greater than that induced by the commercial vaccine.

### 3.5 Viremia detection

The results of PCV2 viremia detection are listed in Table 1. At 7 days post-challenge, viremia was detected in the PBS, PCV2-Cap, PCV-Cap-2-3, and PCV2-CV groups. At 14 days post-challenge, viremia was detected in 5/5 pigs in the PBS group, 2/5 pigs in the PCV2-Cap and PCV-Cap-2-3 groups, and 3/5 pigs in the PCV2-CV group. At 21 days post-immunization, viremia was detected in 5/5 pigs in the PBS group, and in 1/5 pigs in each of the PCV2-Cap, PCV-Cap-2-3, and PCV2-CV groups. At 28 days post-immunization, no viremia was detected in the PCV2-Cap, PCV-Cap-2-3, or PCV2-CV groups, whereas viremia was detected in 4/5 pigs in the PBS group.

**Table 1:** Detection of viremia PCV2 post-challenge results.

Groups	Days of PCV2 post-challenge				
	0	7	14	21	28
PBS	0/5	5/5	5/5	5/5	4/5
PCV2-Cap	0/5	5/5	2/5	1/5	0/5
PCV-Cap-2-3	0/5	5/5	2/5	1/5	0/5
PCV2-CV	0/5	5/5	3/5	1/5	0/5

The results of PCV3 viremia detection are listed in Table 2. At 7 days post-challenge, viremia was detected in 4/5 pigs in the PBS group, and in 3/5 pigs in both the PCV3-Cap and PCV-Cap-2-3 groups. At 14 days post-challenge, viremia was detected in 4/5 pigs in the PBS group, and in 2/5 pigs in both the PCV3-Cap and PCV-Cap-2-3 groups. At 21 days post-immunization, viremia was detected in 3/5 pigs in the PBS group, and in 1/5 pigs in both the PCV3-Cap and PCV-Cap-2-3 groups. At 28 days post-immunization, viremia was detected in 2/5 pigs in the PBS group, whereas no viremia was detected in either the PCV3-Cap or PCV-Cap-2-3 group.

**Table 2:** Detection of viremia PCV3 post-challenge results.

Groups	Days of PCV3 post-challenge				
	0	7	14	21	28
PBS	0/5	4/5	4/5	3/5	2/5
PCV3-Cap	0/5	3/5	2/5	1/5	0/5
PCV-Cap-2-3	0/5	3/5	2/5	1/5	0/5

### 3.6 Histopathological examination

**Figure 5:**
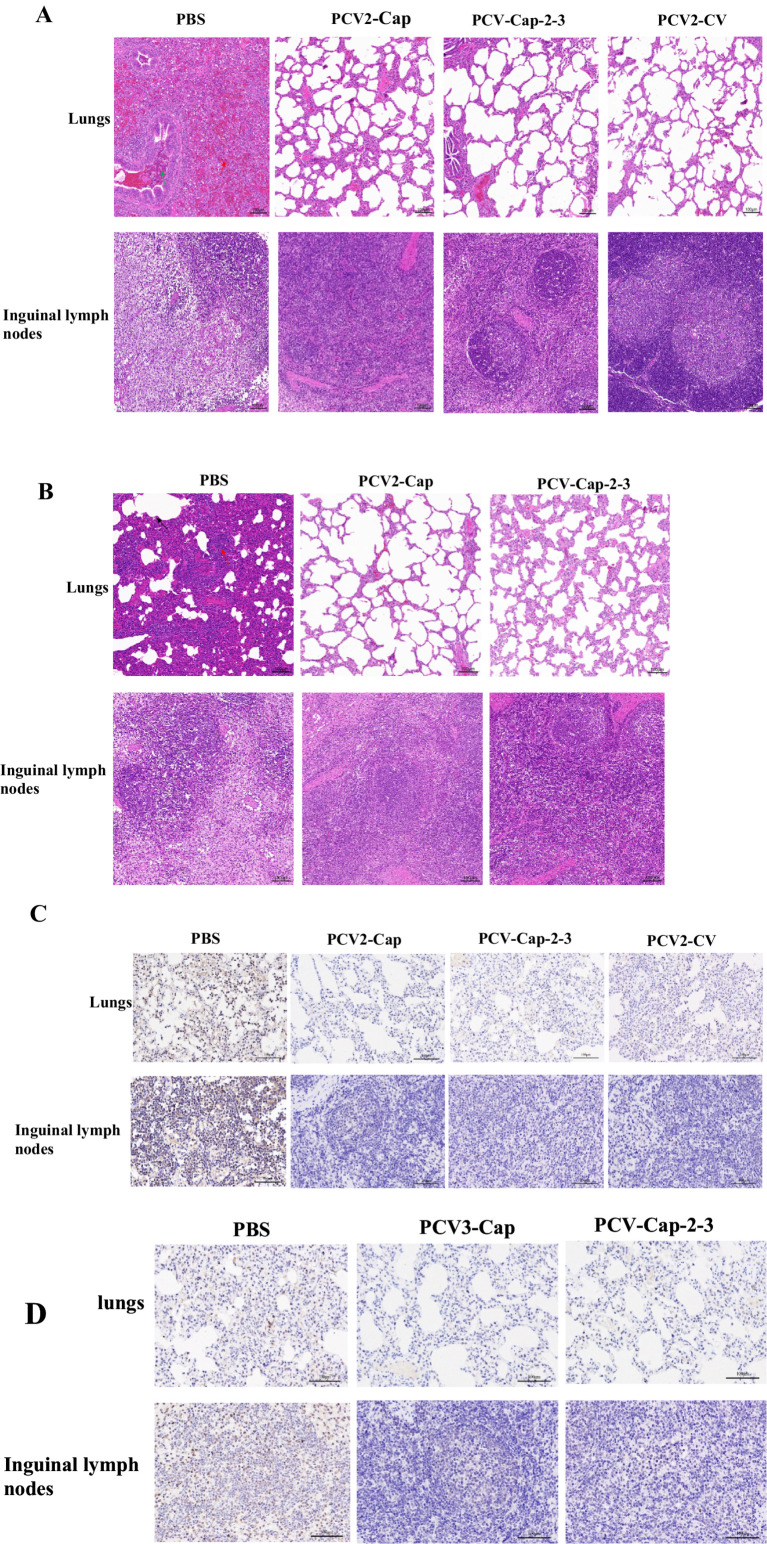
Pathological and immunohistochemical analyses after PCV2/PCV3 challenge (scale bar: 100 μm). **(A,B)** After challenge with PCV2 **(A)** or PCV3 **(B)**. **(C,D)** Immunohistochemical analysis of PCV2-specific **(C)** or PCV3-specific **(D)** antigens.

Following challenge with PCV2, the lung tissue exhibited severe structural abnormalities (Figure 5A), including extensive alveolar atrophy and collapse, parenchymal consolidation, and marked inflammatory cell infiltration and hemorrhage (red arrows). The bronchial lumen contained abundant inflammatory exudates and red blood cells (green arrows). In the PBS-immunized group, the pathological changes were primarily characterized by thickening of the alveolar septa and inflammatory cell infiltration. In contrast, no significant abnormalities were observed in the lungs of the PCV2-Cap-, PCV-Cap-2-3-, or PCV2-CV-immunized groups. In the PBS-immunized group, the inguinal lymph nodes primarily exhibited reduced lymphocyte counts and enlarged interstitial spaces, whereas no significant abnormalities were observed in the other immunized groups.

Following challenge with PCV3, the lung tissue exhibited severe abnormalities (Figure 5B), including extensive alveolar atrophy and collapse, thickened alveolar walls, and parenchymal consolidation. The remaining alveoli were fused and dilated to form pulmonary bullae (black arrows), and extensive inflammatory cell infiltration was evident within the lung parenchyma (red arrows). The PBS-immunized group exhibited severe pathological changes in lung tissue, primarily characterized by severe structural abnormalities, thickened alveolar septa, and inflammatory cell infiltration. In contrast, no significant abnormalities were observed in the lungs of the PCV3-Cap- and PCV-Cap-2-3 immunized pigs. In the PBS-immunized group, the inguinal lymph nodes primarily showed reduced lymphocyte counts and enlarged interstitial spaces, whereas no significant abnormalities were observed in the other groups.

### 3.7 Immunohistochemical detection

PCV2 and PCV3 antigens were detected in the lungs and lymph nodes of pigs in the PBS group following immunization and challenge, presenting as diffuse or granular brown-yellow staining. In contrast, no specific brown-yellow staining was detected in the lungs and lymph nodes of pigs in each experimental groups following immunization and challenge (Figures 5C,D).

## 4 Discussion

PCVs, particularly PCV2 and PCV3, are major viral threats to the global swine industry. PCV2 is the primary causative agent of multiple PCVADs, leading to significant economic losses in the industry (13, 14). Since the discovery and spread of PCV3, the virus has been increasingly associated with various clinical symptoms, such as skin inflammation, porcine dermatitis and nephropathy syndrome, reproductive failure, and multisystemic inflammation, which are highly similar to those caused by PCV2. Furthermore, the global spread of PCV3 and its potential for cross-species transmission have raised significant concerns within the industry (15, 16). The high prevalence of PCV2 and PCV3, coupled with their co-infection patterns, further complicates disease control. Both viruses exhibit high infection rates in pig farms, with a significant number of co-infection cases, suggesting that synergistic infections exacerbate clinical symptoms and pathological damage in pigs (17–19). Hence, vaccination remains the most effective strategy for PCV prevention and control. To the best of our knowledge, the Cap protein is the only PCV structural protein with multiple antigenic epitopes.

In the present study, we analyzed the Cap genes of the predominant PCV2d and PCV3b genotypes. To enhance recombinant protein expression levels and antigenicity while ensuring fusion protein stability, a rigid linker peptide was used to connect the PCV2 and PCV3 Cap proteins. The inserted genes were then optimized. A Kozak sequence was introduced at the 5′ end of the Cap gene to increase protein expression efficiency. Using a baculovirus expression system, PCV2-Cap and PCV3-Cap genes were expressed as fusion constructs in a baculovirus expression vector system, achieving high-level expression. The PCV2-Cap, PCV3-Cap, and PCV-Cap-2-3 bivalent subunit vaccines were prepared and administered to pigs. Vaccine immunogenicity was assessed by evaluating humoral and cellular immune responses against PCV2 and PCV3. The immunoprotective efficacy against PCV2 and PCV3 was evaluated based on clinical symptoms, viremia, and histopathological examination (20, 21).

Both truncated NLS-deleted Cap proteins and full-length Cap proteins have potential for development as subunit vaccines (22–24). Our findings showed that PCV2-Cap, PCV3-Cap, and PCV-Cap-2-3, which were co-expressed without removal of the NLS sequence, could also be expressed in this system. The recombinant proteins exhibited high reactivity, suggesting that Cap proteins expressed from the complete ORF2 sequence more closely resemble the native protein (25). The production of specific and neutralizing antibodies is a key indicator of vaccine-induced humoral immunity. Wang et al. have reported that subunit vaccines expressing fusion proteins of PCV2, PCV3, and PCV4 and multiple Cap proteins effectively induce specific antibodies against all PCV types and neutralize PCV2 and PCV3, highlighting their strong immunoprotective efficacy (24). We successfully constructed baculovirus-based monovalent PCV2-Cap and PCV3-Cap vaccines, as well as a bivalent PCV-Cap-2-3 subunit vaccine. Animal experiments confirmed that all vaccines induced humoral immunity. Notably, the bivalent vaccine induced the production of specific and neutralizing antibodies against both PCV2 and PCV3, with superior immunogenicity against PCV2 compared with that of commercial vaccines, highlighting its promising application potential.

Lymphocyte proliferation assays and cytokine measurements are key indicators of vaccine-induced cellular immunity (20, 26). The Th1 immune response is characterized by IFN-γ secretion, which enhances cellular immunity (27, 28). In contrast, the Th2 immune response is characterized by IL-4 secretion, which enhances humoral immunity (29). Our results indicate that the PCV-Cap-2-3 bivalent subunit vaccine induces both Th1- and Th2-type immune responses, as evidenced by increased secretion of IFN-γ (Th1) and IL-4 (Th2), thereby enhancing cellular and humoral immunity, respectively. The lymphocyte proliferation assay further confirmed that the PCV-Cap-2-3 bivalent subunit vaccine induced cellular immune responses against both PCV2 and PCV3.

Challenge protection following vaccination is a key indicator of vaccine efficacy (30). The predominant circulating strains of PCV2 and PCV3 are PCV2d and PCV3b, respectively. Previous studies in our laboratory involving strain isolation and pathogenicity testing in mice and pigs have demonstrated that PCV2d and PCV3b can cause viremia and pathological lesions. PCV2 or PCV3 infection resulted in structural abnormalities in the lung tissue, with alveolar atrophy and collapse, lung parenchymal consolidation, and marked inflammatory cell infiltration with hemorrhage. Additionally, the number of lymphocytes decreased while intercellular spaces widened, consistent with the results of Zheng et al. (31). Following PCV2 challenge, the PBS-immunized group exhibited viremia and histopathological damage, whereas the PCV2-Cap, PCV-Cap-2-3, and commercial PCV2 vaccine groups showed no significant pathological damage; furthermore, viremia was significantly reduced, consistent with the results of Yu et al. (32).

In the present study, since no continuous cell line is currently available for the isolation and purification of PCV3, our laboratory adopted the PCV3b strain previously passaged in primary porcine kidney cells as the challenge strain for evaluating the protective efficacy of the vaccine, aiming to further investigate the protective efficacy of PCV3 vaccines. Following PCV3 challenge, low viral loads were detected in PCV3-positive samples during viremia monitoring. Histopathological examination revealed PCV2-like features, such as alveolar interstitial thickening and inflammatory cell infiltration. The vaccinated groups exhibited slightly less tissue damage than the PBS group, which may be attributable to defects in the isolated PCV3 strain, such as instability and low viral load. Attempts to propagate PCV3 using ST, 3D4/21, PK-15, Marc145, Vero, and CRFK cells were unsuccessful, and PCV3 was passaged in primary porcine kidney cells for only five generations. Future experiments will improve PCV3 isolation to obtain a strain capable of stable passage in cells for challenge protection tests. These results were compared with those from the PCV3 challenge protection test to further validate our findings. During immunization, vaccines formulated with GEL02 adjuvants yielded low antibody levels after a single dose, and two immunizations were required to achieve the desired antibody levels, indicating potential for optimization. The experimental results suggest that the purified protein vaccine has slightly better efficacy, which may be because most PCV2 baculovirus vaccines on the market are whole-virus inactivated vaccines with lower protein purity than subunit vaccines. However, owing to the high cost of rod-shaped virus vaccines, future studies should focus on cell culture optimization and antigen purification techniques to determine whether a single immunization can achieve equivalent efficacy, which would enhance vaccine efficacy and improve its prospects for clinical applications.

In conclusion, the rescued recombinant baculoviruses Baculovirus-PCV2-Cap, Baculovirus-PCV3-Cap, and Baculovirus-PCV-Cap-2-3 exhibited successful protein expression in cells and allowed protein purification. The resulting PCV-Cap-2-3 bivalent subunit vaccine, prepared with the GEL02 adjuvant, induced strong humoral and cellular immune responses in pigs. It effectively reduced the effects of PCV2 and PCV3 challenge and mitigated pathological damage to lung and lymphoid tissues. No significant differences in performance were observed compared with existing commercial PCV2 vaccines, indicating that the developed PCV-Cap-2-3 subunit vaccine is a viable candidate for further development.

## Data Availability

The raw data supporting the conclusions of this article will be made available by the authors, without undue reservation.
